# Lake Keuka Medical Association

**Published:** 1903-09

**Authors:** 


### Lake Keuka Medical Association.

THE midsummer medical society is so unique that we feel sure a little space set apart for the consideration of such an association will not be appropriated amiss. The Lake Keuka meeting which is appointed for August has lately been held at Grove Springs, and we publish the minutes elsewhere. This association was organised in 1900, by some of the younger physicians residing in the counties adjacent to the lovely and picturesque region of the beautiful lake that gives its name to the society.

The meeting recently held under the presidency of Dr. Arthur M. Booth, of Elmira, was a credit in every way to the officers who managed its affairs, and to all who participated in the proceedings. The program contained a group of well selected papers that were presented with sustained interest, from the beginning to the end,—from the president's address to the election of officers. Dr. Booth's address dealt with surgical intervention in the perforative complications of typhoid fever, and was a masterful presentation of the subject. We expect to print it in a future number of the JOURNAL, as well as all of the papers read at the meeting.

The number in attendance was 75, which bespeaks the interest taken in the association. Dr. Augustin H. Goelet came all the way from New York to participate in the proceedings, and he contributed to the interest of the occasion in a marked degree, remaining until the last. Also there were physicians present from Syracuse, Rochester, Buffalo and Elmira, besides the usual quota from the counties bordering on Lake Keuka, so that there was no lack of an attentive audience to the reading of any paper.

The Lake Keuka Club threw open its doors to the members and visitors in a most hospitable manner. A smoker was held at the club on Tuesday evening,—the evening of the first day,—which was attended by the entire association, and proved a most enjoyable occasion. The election of officers resulted as follows: president, A. L. Beahan, Canandaigua; vice-president, P. L. Alden, Hammondsport; secretary and treasurer, W. W. Smith, Avoca. Dr. Smith has held the double office of secretary and treasurer from the beginning, and to his energy and efficiency the association in large measure owes its successful career. The meeting closed as it began, with enthusiasm, and everybody in attendance declared intentions to come next year.

### URBANA WINE COMPANY.

After the meeting adjourned all present, including many ladies, boarded the steam yacht "Earl," Captain A. D. B. Grimley, commander, which had been at the disposal of the association during the meeting, and by invitation and under the escort of Colonel A. J. Switzer, secretary of the Urbana Wine Company, a visit was paid to the famous cellars of that great wine producing industry. Col. Switzer came across the lake to Grove Springs to receive the visitors and conduct them through the vaults. He assigned to each of the several groups into which the party was divided a guide, and each person was supplied with a lighted candle. The experience was interesting and the scene weird. Groping through the narrow avenues of the deep vaults with lighted torches, more than 800,000 bottles of sparkling wine were inspected in all stages of the maturing and settling process; also, more than 300,000 gallons of still wine was seen in containers, holding from 1,000 to 6,000 gallons, the average being about 2,000 gallons a cask.

This company was organised in 1865 and has steadily grown to be one of the largest producers of wine in the world. It markets its output in all quarters of the globe and is noted for the perfection and quality of its wines, both sparkling and still. The officers are: president, W. E. Hildreth; vice-president, M. M. Acker; treasurer, J. S. Reynolds; secretary, A. J. Switzer; general manager, James Neal. Colonel Switzer has been the executive officer of the company for more than twenty years, and is known far and wide for his capacity as a man of affairs, as well as for his genial temperament and affability of manner.

The Urbana Wine Company puts up a sparkling wine in half pint bottles specially adapted for medicinal uses, which fact is an important one for clinicians to remember. The charging or "disgorging" of corks, as it is called, is an interesting process, and owes its success to the clever inventor who has devised appropriate machines for the purpose, whereby there is no loss in substituting the cork of the cellar for the cork of commerce,—all of which should be witnessed to be appreciated. All the guests were greatly interested in what they had seen and expressed in terms their appreciation of the courtesy,—the Urbanity we might say,—of the officers of the company who entertained the visitors so royally.

